# The Importance of Phase Composition for Corrosion Resistance of Borided Layers Produced on Nickel Alloys

**DOI:** 10.3390/ma13225131

**Published:** 2020-11-13

**Authors:** Natalia Makuch

**Affiliations:** Institute of Materials Science and Engineering, Poznan University of Technology, Pl. M. Skłodowskiej-Curie 5, 60-965 Poznan, Poland; natalia.makuch@put.poznan.pl

**Keywords:** boriding, nickel borides, chromium borides, corrosion resistance, pitting corrosion

## Abstract

The plasma paste boriding process was used for production of the borided layers on pure nickel and nickel-chromium alloys. The produced layers consisted of nickel borides only (in the case of nickel) or a nickel and chromium borides mixture (in the case Ni–Cr alloys). The objective of this investigation was to indicate the importance of the presence of chromium for corrosion resistance of non-borided alloys, as well as to indicate the influence of phase composition of borided layers on their corrosion resistance. Pure nickel was characterized by higher corrosion resistance, in comparison to the nickel-based alloys. Increased chromium content in nickel alloys resulted in their high susceptibility for pitting corrosion. All borided samples were characterized by higher corrosion resistance than the non-borided samples. However, the phase composition of borided layers influenced their corrosion resistance. Due to the microstructure which consisted of one type of borides (nickel borides), borided nickel had the highest resistance to corrosion, whereas the presence of chromium borides in layers produced on nickel-chromium alloys caused a decrease in corrosion resistance.

## 1. Introduction

Nickel alloys are commonly known for their attractive properties, which led to using them in such areas as, for example, the chemical engineering industry, the petroleum industry or for turbine construction. The main advantageous properties of Ni-based alloys are as follows: resistance to aggressive acidic and alkaline solutions, resistance to oxidation at high temperature, and creep resistance. Unfortunately, the use of nickel alloys is limited due to their poor wear resistance, high friction and strong tendency to galling. Obviously, the production of surface layers causes an increase in the tribological properties of pure nickel and its alloys. Different boriding methods are dedicated to improve the wear resistance of nickel alloys: powder boriding with the usage of agents without SiC [1,2,3,4], gas boriding in a N_2_–H_2_–BCl_3_ atmosphere [5,6,7,8,9,10], electrochemical boriding in molten borax [11] or paste boriding in Ekabor paste [12]. In recent years, the plasma boriding technique was applied to the treatment of steels [13,14,15] and titanium alloys [16,17]. The production of borided layers on nickel alloys is also possible with the use of the plasma boriding method [18]. The process was carried out on pure nickel and nickel-chromium alloys. It was found that the increase in chromium concentration in the substrate material resulted in the reduction in the borided layer thickness. The higher depth (54.24 µm) of the layer was obtained for chromium-free Nickel 201. An increase in the Cr content in Inconel^®^600 and Nimonic^®^80A alloys resulted in a layer thickness reduction, to 44.41 and 41.31 μm, respectively. The differences in the chromium concentration in the substrate materials influenced the phase composition of the produced layers. In the case of chromium-free substrate material the produced layer only contained nickel borides. Simultaneously, the presence of chromium in Inconel^®^600 and Nimonic^®^80A alloys resulted in the formation of multicomponent layers consisting of nickel borides and chromium borides. The mechanical properties were dependent on the chromium content in the base material and the phase composition of the produced layers. The lowest hardness (17.97 GPa) and Young’s modulus (217.89 GPa) were measured in the boride layer containing only nickel borides. The chromium presence in Inconel^®^600 and Nimonic^®^80A alloys resulted in higher hardness, 19.41 and 22.96 GPa, respectively. Simultaneously, these layers were characterized by a higher Young’s modulus, 274.32 and 291.95 GPa, respectively. Obviously, the increase in hardness and Young’s modulus of borided layers produced on nickel-chromium alloys was the reason for higher brittleness of these layers. The plasma paste borided layer, in which only nickel borides were identified, was characterized by the highest average fracture toughness of 1.477 MPa·m^1/2^. The average fracture toughness measured in the boride layer produced on Inconel^®^600 alloy was equal to 0.719 MPa·m^1/2^. Simultaneously, the further increase in chromium content in the Nimonic^®^80A alloy (up to 19.52 wt.%) resulted in the lowest average fracture toughness (*K*_c_ = 0.534 MPa·m^1/2^). Based on the results presented in paper [18] it was found that the chromium concentration in nickel alloys was responsible for the differences in obtained microstructure, thickness, hardness and brittleness of plasma-paste borided layers. The plasma-assisted boriding method became interesting for industrial application because of the diminishing of gas consumption at low pressure. Moreover, during the process the treated surface and the gases used are activated under glow discharge conditions, therefore the temperature of the process is diminished. Improvement in hardness and tribological properties of borided nickel alloys must be accompanied by their property of good resistance in corrosive environments.

Generally, nickel and its alloys are characterized by a high corrosion resistance. The main alloying element which improves the corrosion resistance of nickel alloys in oxidizing environments is chromium. Chromium also confers resistance to sulfur compounds. The addition of iron caused increased corrosion resistance in sulphuric acid in concentrations above 50% [19,20]. However, the addition of chromium, titanium and iron leads to obtaining multi-phase microstructure with precipitation of carbides and nitrides at the grain boundaries [21,22,23]. The precipitation of chromium-rich carbides at grain boundaries could have a profound effect on the susceptibility of Inconel^®^600 alloy to intergranular corrosion [24,25]. However, the influence of boriding on corrosion resistance of different nickel alloys was still not recognized. Corrosion resistance of borided layers produced on pure nickel and Nimonic 90 alloy was reported in the paper [12]; only in this paper [12] was a comparison reported between the corrosion resistance of the borided layers produced on pure nickel and Nimonic^®^90 alloy. Immersion corrosion tests were performed in an artificial seawater solution. The differences in phase composition of the produced layers were observed. Boriding of pure nickel resulted in formation of a single-phase layer. Whereas borided Nimonic^®^90 alloy contained nickel borides and chromium borides, it was found the borided Nimonic^®^90 alloy had a lower corrosion resistance than the borided pure nickel. Differences in the phase composition of both layers were given as the probable cause of this situation. However, no detailed information and explanations were provided.

For this reason, in the present study a comparison was made of the corrosion resistance of borided layers produced on pure nickel and Ni–Cr alloys. In addition, the comparison between corrosion resistance of borided and non-borided samples was also presented. The plasma paste boriding process was applied to production of the boride layers on the nickel-based alloys, which differed in chromium content: Nickel 201 (0 wt.% Cr), Inconel^®^600 (15.72 wt.% Cr), and Nimonic^®^80A (19.52 wt.% Cr). Corrosion resistance was determined based on the polarization curves and topography of the corroded surfaces. The presented results allowed determining the influence of the phase composition of the borided layers on their corrosion resistance. The detailed description and explanation of obtained differences in corrosion resistance in correlation with phase composition of tested samples, are the novelty of present work.

## 2. Experimental Procedure

### 2.1. Material

Substrate materials used for this study were pure Nickel 201 and nickel-chromium alloys which differed in chromium concentration: Inconel^®^600 alloy and Nimonic^®^80A alloy. The concentrations of elements in each material are presented in Table 1.

### 2.2. Boriding

The plasma pastes boriding (PPB) technique was applied for production of borided layers on pure nickel and Ni–Cr alloys. The equipment used for the process was presented in an earlier paper [18]. The following process parameters were used: temperature of 800 °C (1073 K), duration of 180 min, constant pressure of 50% Ar-50% H_2_ gas mixture of 5 mbar (500 Pa). The source of boron was a paste which contained borax (Na_2_B_4_O_7_).

### 2.3. Microstructure Characterization

The standard metallographic preparation was used in order to prepare the samples for microscopic observations. Etching with Marble’s reagent allowed detailing microstructure characterization. The observations were carried out with the use of scanning electron microscopy (SEM) Tescan Vega 5135 (TESCAN, Poznan, Poland). The phases occurring in the borided layers were identified with the use of a PANalytical EMPYREAN X-ray diffractometer (TESCAN, Poznan, Poland) using Cu Kα radiation.

### 2.4. Corrosion Resistance Evaluation

A potentiodynamic anodic polarization test in 3.5% NaCl solution was used for evaluation of corrosion resistance. The investigations were carried out using an ATLAS 0531 EU&IA (Atlas Solich, Poznan, Poland) device equipped with Atlas Lab v 2.24 software. A three-electrode cell system containing a counter electrode (platinum electrode), a reference electrode (saturated calomel electrode), and a working electrode (tested sample) were used for the experiments. The exposed surface was equal to 50 mm^2^ and was kept immersed in the 3.5% NaCl solution at a constant temperature of 22 °C. The specimens were polarized in the anode direction from the potential −1.5 V up to 1.5 V, with a change rate potential equal to 0.5 mV/s. From the recorded polarization curves (*E-log I*) the following quantities could be determined: corrosion potential *E_corr_*, corrosion current density *I_corr_*, primary passivation potential *E_pp_*, Flade potential *E_F_,* transpassive potential *E_tp_*, secondary passivity potential *E_sp_*, oxygen evolution potential *E_oe_* and the passivation current density.

**Figure 1:**
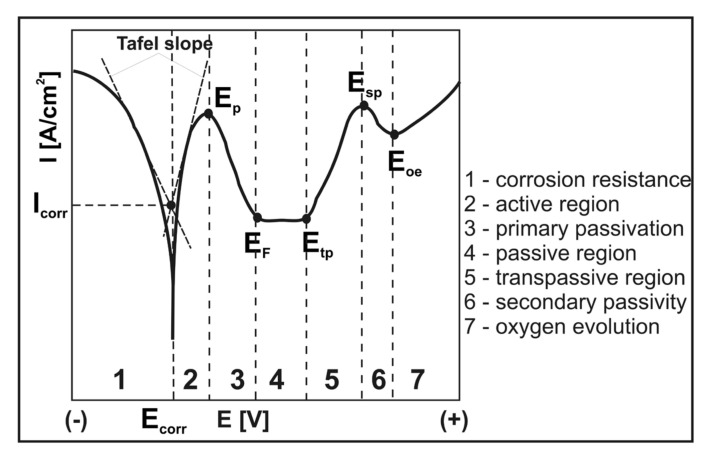
The scheme of a hypothetical polarization curve. *E_corr_*—corrosion potential; *I*_corr_—corrosion current density; *E_pp_*—the primary passivation potential; *E_F_*—the Flade potential; *E_tp_*—the transpassive potential; *E_sp_*—the secondary passive potential; and *E_oe_*—the oxygen evolution potential.

The exemplary kinetics of the electrochemical corrosion process could be characterized by a hypothetical polarization curve (Figure 1). The results are plotted as a diagram, which presents the dependence of electric potential *E* of an electrode on current density *I*. The two main parameters could be calculated based on the polarization curve: corrosion potential *E_corr_* and corrosion current density *I_corr_*. The corrosion current density *I_corr_* was obtained by extrapolating the currents in the two Tafel regions. The Tafel slopes of polarization curves defined the small or large difficulty of the cathodic and anodic processes. Based on Figure 1, in the area where the anodic current increases rapidly, localized corrosion is initiated. The more noble (+) potential, the less susceptibility of the analyzed alloy to initiation of localized corrosion. Some other parameters could be also determined from the potentiodynamic polarization curves. In Figure 1, the following parameters are designated: the primary passivation potential *E_pp_* (the positive potential at which passive surface layer is formed), the Flade potential *E_F_* (the potential at which a metal or alloy undergoes from a passive state to an active state), the transpassive potential *E_tp_* (corresponding to the end of the passive region and to the initial point of anodic evolution of oxygen), the secondary passivation potential *E_sp_*, and oxygen evolution potential *E_oe_*. The point *E_tp_* on the polarization curves determined the end of the passive state of the metallic material. For some combinations of material and aggressive medium (e.g., aluminum alloys in salt water), this sudden increase in current (higher up *E_tp_*) could result from pitting corrosion (localized breakdown in passivity), whereas for others it might be connected with transpassive dissolution. Above the *E_tp_* value, a quick dissolution of the metallic material and the oxidation of passive layer started on the surface. This process could cause the creation of the oxide layer, containing metals on higher oxidation degree, or total dissolution of the passive layer. In case of a stronger polarity, at the *E_sp_* point, the secondary passivation area could occur, and the evolution of oxygen could be observed above the *E_oe_* point.

After the corrosion resistance test the macroobservations of exposed surfaces was carried out. Moreover, the 3D surface topography and line profile across the tested area were investigated with use of the Profilm3D^®^ (Filmetrics, Lowicz, Poland). This profilometer uses white light interferometry (WLI) to measure surface profiles and roughness. The following parameters were determined: *R_a_*, *R_q_*, *R_p_*, *S_a_*, *S_q_, S_p_*, and *S_z_*. Amplitude parameters are the most important parameters characterizing the surface topography in respect of the surface deviations. The arithmetic average height *R_a_* is defined as the average absolute deviation of the roughness irregularities from the mean line. The extension of the *R_a_* parameter to a surface is *S_a_*. The root mean square roughness *R_q_* represents the standard deviation of the distribution of surface heights in the line profile, whereas *S_q_* represents the root mean square value in relation to the surface. The maximum peak height was represented by *R_p_* and *S_p_*, with respect to line roughness and area roughness, respectively. The maximum height measured in the tested area was expressed as *S_z_*.

## 3. Results and Discussion

### 3.1. Microstructure

**Figure 2:**
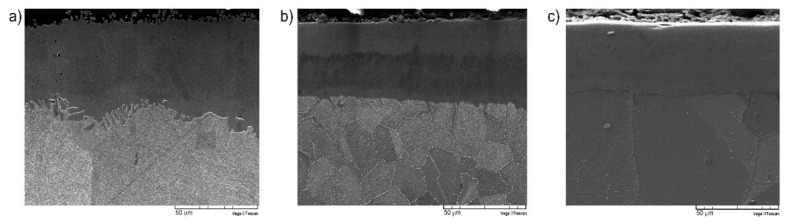
The SEM image of borided layers produced on: (**a**) Nickel 201, (**b**) Inconel^®^600 alloy and (**c**) Nimonic^®^80A alloy.

**Figure 3:**
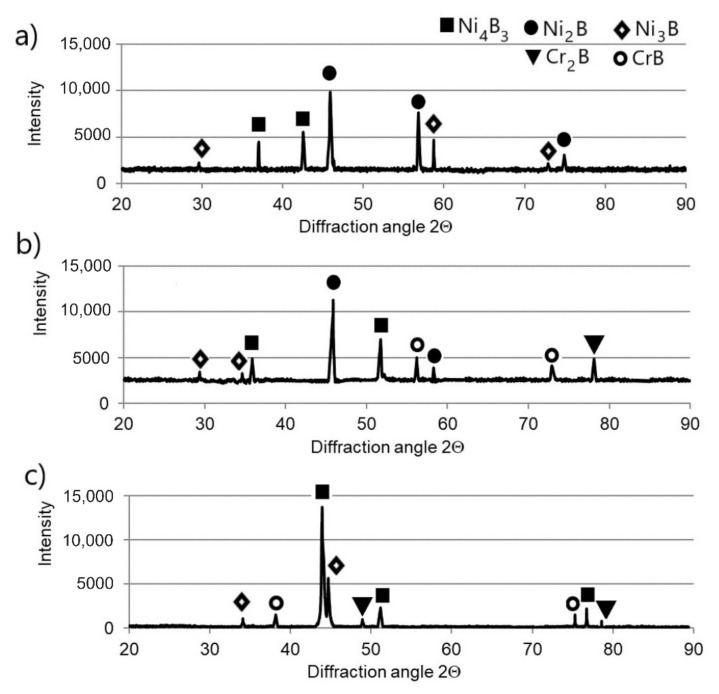
XRD patterns of borided layers produced on: (**a**) Nickel 201, (**b**) Inconel^®^600 alloy and (**c**) Nimonic^®^80A alloy.

The microstructure and phase composition of the borided layers strongly influenced their properties. The differences in chromium content in nickel alloys used in this study caused the production of layers which differed in thickness and phase composition. The scanning electron microscopy (SEM) images of plasma paste borided layers produced on pure Nickel 201 and nickel-chromium alloys (Inconel^®^600, Nimonic^®^80A) are shown in Figure 2, whereas the diffraction patterns of borided samples are presented in Figure 3. The uniform and porous-free layers are obtained on each substrate material. These two factors could advantageously influence the corrosion resistance, because the presence of porosity, as well as the heterogeneity of layers’ thicknesses decreases the corrosion resistance. It was expected that the higher chromium concentration in substrate material caused diminished borided layer thickness. Therefore, the highest average thickness of 54.24 μm was measured for the borided layer produced on Nickel 201. The high chromium content in the Inconel^®^600 and Nimonic^®^80A alloys resulted in a reduction of the layer thickness to 44.41 and 41.31 μm, respectively. The peaks corresponding to the Ni_2_B and Ni_3_B borides were identified for borided Nickel 201 (Figure 3a). It was expected that the high chromium concentration in Inconel^®^600 and Nimonic^®^80A alloys would also cause the formation of chromium borides. This was confirmed by the XRD patterns shown in Figure 3b,c, respectively. In both layers, in addition to nickel borides, the CrB and Cr_2_B phases were identified.

### 3.2. Corrosion Resistance

**Figure 4:**
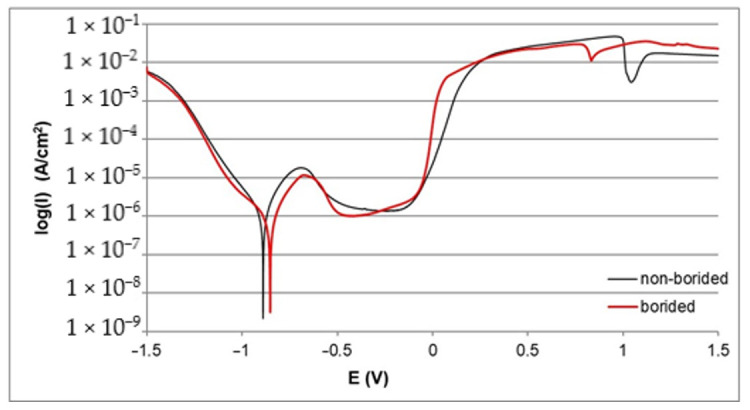
Polarization curves recorded during the corrosion resistance test in a 3.5% NaCl solution for borided and non-borided Nickel 201.

The corrosion resistance of borided and non-borided samples was determined on the basis of polarization curves. The electrochemical parameters *I_corr_* and *E_corr_* were determined and are compiled in Table 2. First, the comparison between borided and non-borided Nickel 201 is shown in Figure 4. Both polarization curves follow a similar course with visible regions of passivation and transpassivation. Moreover, the location of the passive region on the curve is similar in both cases. However, a higher width of corrosion resistance region is observed for borided Nickel 201. For this reason, the value of the corrosion potential was also higher for the borided sample (*E_corr_* = −0.889 V for the non-borided sample and *E_corr_* = −0.853 V for the borided sample). The average value of the passivation current density (*I_pass_*=1.5·10^−6^ A/cm^2^) was similar, as well. The results obtained for Nickel 201 show that the presence of the borided layer causes slightly diminished corrosion. Both samples indicate a tendency to passivation in the solution that was used.

**Figure 5:**
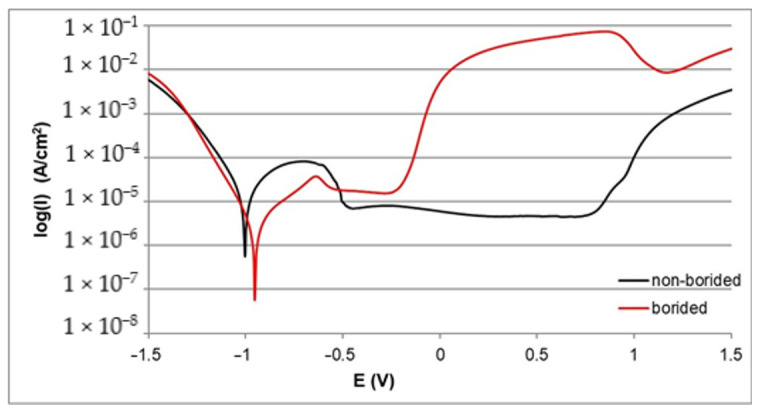
Polarization curves recorded during the corrosion resistance test in a 3.5% NaCl solution for borided and non-borided Inconel^®^600 alloy.

The polarization curves recorded for borided and non-borided Inconel^®^600 alloy are presented in Figure 5. The differences between both curves are visible. The width of the corrosion resistance region was higher for the borided sample in comparison to the non-borided sample. The higher value of the corrosion potential (*E_corr_* = −0.953 V) is characteristic of plasma paste borided Inconel^®^600 alloy. The non-borided sample demonstrated a lower value of *E_corr_* (−1.002 V). The lower corrosion current density *I_corr_* (1.1·10^−6^ A/cm^2^) was measured for borided Inconel^®^600 alloy. The non-borided sample was characterized by a higher value of *I_corr_* (9.8·10^−6^ A/cm^2^). These two quantities indicated that the plasma paste borided sample shows a higher corrosion resistance in 3.5% NaCl solution. Comparing the width of the passive region, the non-borided Inconel^®^600 alloy was found to be passive over a wide potential range (from −0.489 V to 0.762 V) before transpassive dissolution at 0.762 V occurred, whereas the borided sample was characterized by a reduced width of the passive region, ranging from −0.508 to 0.237 V. These results indicate that the non-borided Inconel^®^600 alloy has a higher susceptibility to passivation in 3.5% NaCl solution; non-borided Inconel^®^600 alloy also has a characteristic lower average passivation potential.

**Figure 6:**
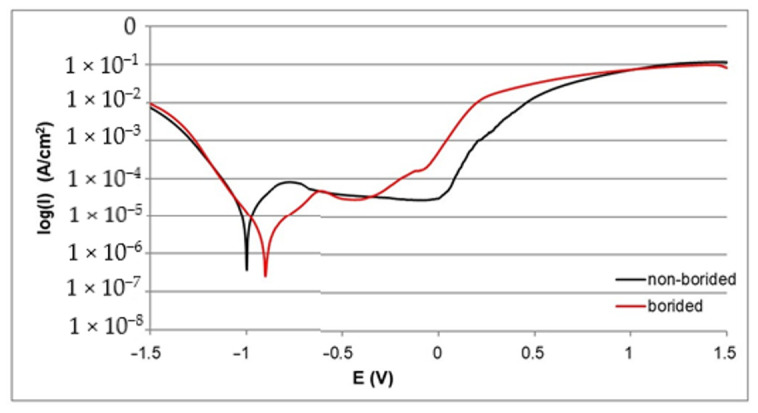
Polarization curves recorded during the corrosion resistance test in a 3.5% NaCl solution for borided and non-borided Nimonic^®^80 alloy.

The polarization curves recorded for the borided and non-borided Nimonic^®^80A alloy are presented in Figure 6. In this case, the highest difference between the width of the corrosion resistance region is visible. For this reason, the plasma paste borided Nimonic^®^80A alloy is characterized by a higher value of the corrosion potential (*E_corr_* = −0.902 V). The non-borided sample demonstrates a lower value of *E_corr_* = −1.003 V. The lower corrosion current density *I_corr_* (1.9·10^−6^ A/cm^2^) was measured for the borided Nimonic^®^80A alloy. The non-borided sample is characterized by a higher value of *I_corr_* (9.7·10^−6^ A/cm^2^). When comparing the width of the passive region, it is concluded that the non-borided Nimonic^®^80A alloy is in a passive state over a wide range of potential (from −0.687 V to 0.028 V), whereas the borided sample is characterized by a lower range of passive state, which extends from −0.505 to −0.085 V. These results indicate a higher susceptibility to passivation in 3.5% NaCl solution of the non-borided Nimonic^®^80A alloy.

In general, all the borided samples were characterized by higher resistance to corrosion in 3.5% NaCl solution in comparison with the non-borided samples. However, the comparison of the polarization curves, as well as the electrochemical parameters listed in Table 2, indicate some differences in the corrosion behavior of the samples. The highest *E_corr_* value and simultaneously the lowest *I_corr_* value were characteristic of the plasma paste borided Nickel 201. This situation required explanation. Nickel 201 does not contain chromium in its chemical composition. Therefore, the borided layer consisted only of nickel borides (Ni_2_B, Ni_3_B, Ni_4_B_3_). Whereas, in the case of nickel-chromium alloys (Inconel^®^600, Nimonic^®^80A) due to the high Cr content, the produced borided layers consisted of a mixture of nickel borides (Ni_4_B_3_, Ni_3_B, Ni_2_B) and chromium borides (CrB, Cr_2_B). The multicomponent character of these layers could be the reason for the formation of corrosion micro-cells. The privileged areas for micro-cells formation were those with different types of borides: nickel borides and chromium borides. These phases showed different physical and chemical properties, as well as the electrochemical potential. As a result of these differences, the microstructure of the boride layers formed on nickel-chromium alloys (Inconel^®^600, Nimonic^®^80A) was partitioned into the anode region and cathode region. Therefore, during potentiodynamic corrosion test, micro-cells were formed between the different types of borides.

It should also be noticed that the non-borided samples differed in behavior in a corrosive environment. The highest corrosion resistance was characteristic of pure nickel, whereas the nickel-chromium alloys were characterized by diminished corrosion potential *E_corr_*. Simultaneously, their corrosion current density *I_corr_* was higher. The important information was also the width of passive region. Due to the high content of chromium Inconel^®^600 and Nimonic^®^80A alloys were more susceptible to passivation in 3.5% NaCl solution.

**Figure 7:**
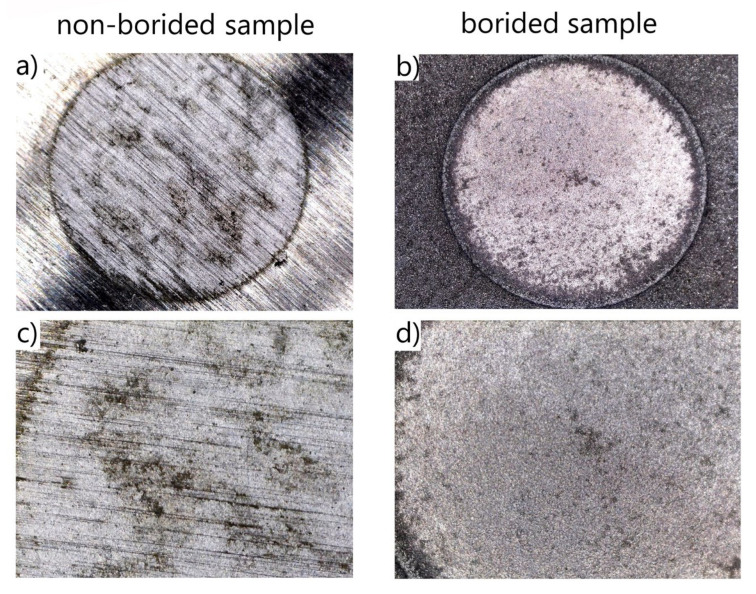
Macroscopic images of the corroded surfaces of non-borided (**a**,**c**) and borided (**b**,**d**) Nickel 201 after the corrosion resistance test in a 3.5% NaCl solution.

After the corrosion resistance tests were finished, the macrostructure of the corroded surfaces was analyzed. The corroded surfaces of borided and non-borided Nickel 201 (Figure 7) were characterized by the presence of a passivating oxide film. However, the corroded surface of non-borided Nickel 201 contained minor corrosion pits that were symptoms of localized corrosion. The corroded surface of borided Nickel 201 could also be classified as a passivated oxide film with local pits of small dimensions. It is well known that nickel alloys are susceptible to pitting corrosion in a corrosive environment containing chloride anions. For this reason, the minor corrosion pits were detected on the surface after the potentiodynamic test in NaCl solution.

**Figure 8:**
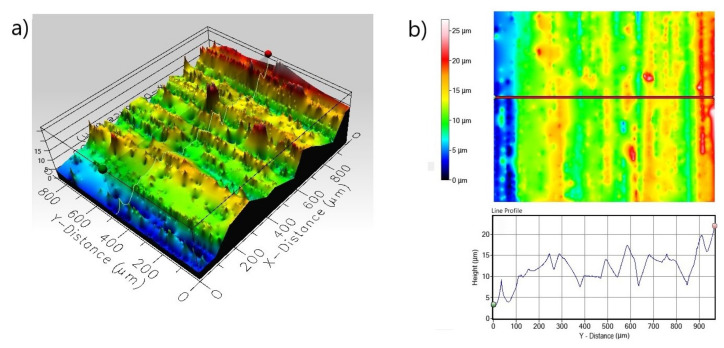
3D profile (**a**) and 2D profile (**b**) of the corroded surface of non-borided Nickel 201 after the corrosion resistance test in a 3.5% NaCl solution.

**Figure 9:**
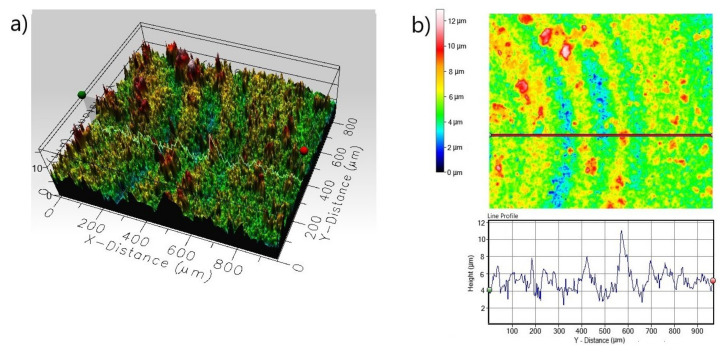
3D profile (**a**) and 2D profile (**b**) of the corroded surface of borided Nickel 201 after the corrosion resistance test in a 3.5% NaCl solution.

The presence of small pits on the surface can also be dangerous because small pits can be very deep and can penetrate deep into the substrate material. Therefore, the 3D and 2D surface profiles of the corroded surfaces were carried out. The roughness parameters determined from line profile and surface profile are presented in Table 3. In the case of non-borided Nickel 201 (Figure 8) the surface is characterized by a roughness resulting from the mechanical treatment performed as a preparation stage before the corrosion test. The measured *R_a_* and *S_a_* were equal to 0.485 and 2.672 µm, respectively. The deep corrosion pits were not identified. The corroded surface of borided pure nickel (Figure 9) show a more uniform profile with lower roughness (*R_a_* = 0.352 µm, *S_a_* = 1.127 µm). Similarly, no deep corrosion pits have been identified. The maximum height *S_z_* measured in the tested area was equal to 11.892 µm.

**Figure 10:**
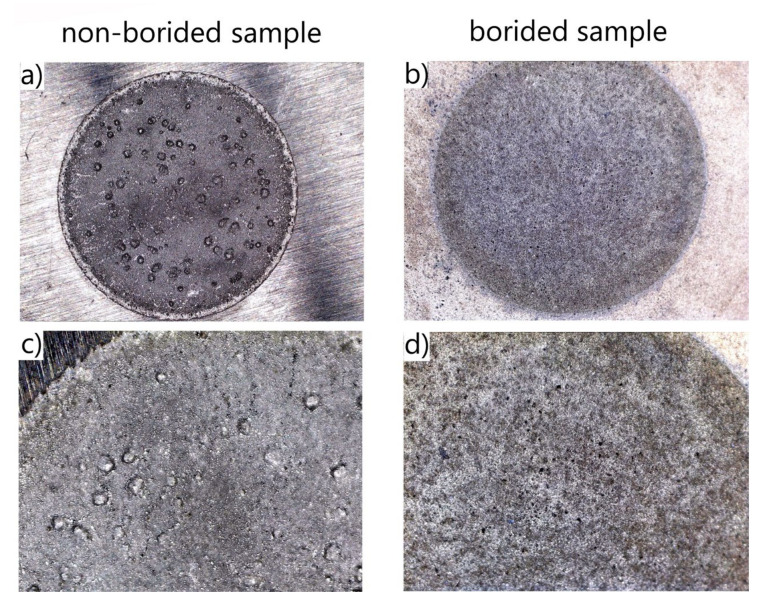
Macroscopic images of the corroded surface of non-borided (**a**,**c**) and borided (**b**,**d**) Inconel^®^600 alloy after the corrosion resistance test in a 3.5% NaCl solution.

**Figure 11:**
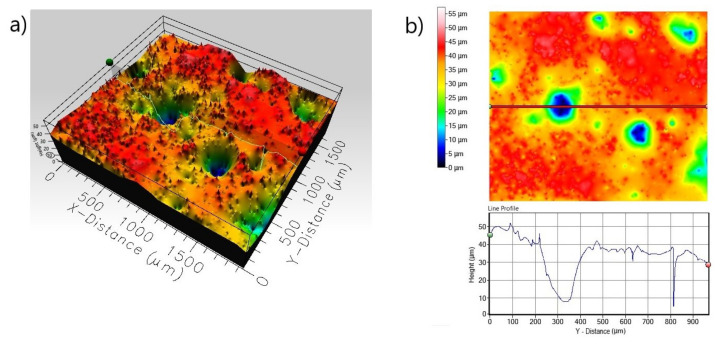
3D profile (**a**) and 2D profile (**b**) of the corroded surface of non-borided Inconel^®^600 alloy after the corrosion resistance test in a 3.5% NaCl solution.

**Figure 12:**
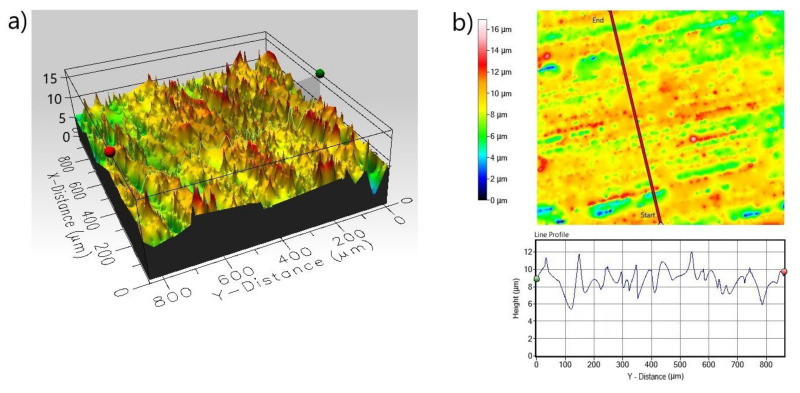
3D profile (**a**) and 2D profile (**b**) of the corroded surface of borided Inconel^®^600 alloy after the corrosion resistance test in a 3.5% NaCl solution.

In the case of plasma paste borided and non-borided Inconel^®^600 alloy (Figure 10) both surfaces are characterized by the presence of a passivating oxide film. However, the intense localized corrosion with the characteristic pits was detected on the corroded surface of the non-borided sample. The corroded surface of plasma paste borided Inconel^®^600 alloy can be classified as a passivated oxide film without local pits. The 3D and 2D profiles of corroded surface were carried out for non-borided and plasma paste borided Inconel^®^600 alloy and are presented in Figure 11 and Figure 12, respectively.

The 3D profile (Figure 11a) of the non-borided sample reveals a strong pitting corrosion attack; a high depth of pit is visible on the line profile obtained from the scan of the corroded surface (Figure 11b). The maximum penetration depth of a single corrosion pit is represented by a maximum peak height *S_p_* = 58.144 µm; the width of a single corrosion pit exceeds 100 µm. The corroded surface of the plasma paste borided Inconel^®^600 alloy is characterized by more uniform profile (Figure 12) with a lower roughness (*R_a_* = 0.253 µm, *S_a_* = 1.191 µm) in comparison to the non-borided sample (*R_a_* = 1.121 µm, *S_a_* = 7.779 µm). Based on the 3D profile and line profile (Figure 12) the deep corrosion pits are not identified. The standard deviation *S_q_* of the distribution of surface heights in the surface area profile was equal to 1.534 and 10.812 µm, for the borided and non-borided sample, respectively.

**Figure 13:**
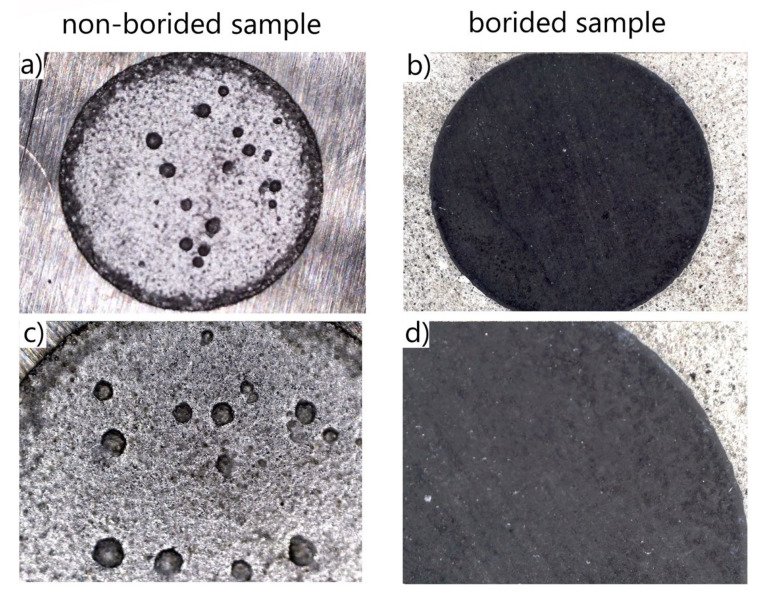
Macroscopic images of the corroded surface of the non-borided (**a**,**c**) and borided (**b**,**d**) Nimonic^®^80A alloy after corrosion resistance test in a 3.5% NaCl solution.

The macroscopic images of the corroded surfaces obtained on the non-borided and borided Nimonic^®^80A alloy are presented in Figure 13. Both corroded surfaces are characterized by the presence of a passivating oxide film. However, the intensive localized corrosion with the characteristic high-dimensioned pits can be observed on the surface of the non-borided sample, whereas the corroded surface of the borided sample is free from the visual effects of pitting corrosion (Figure 13).

**Figure 14:**
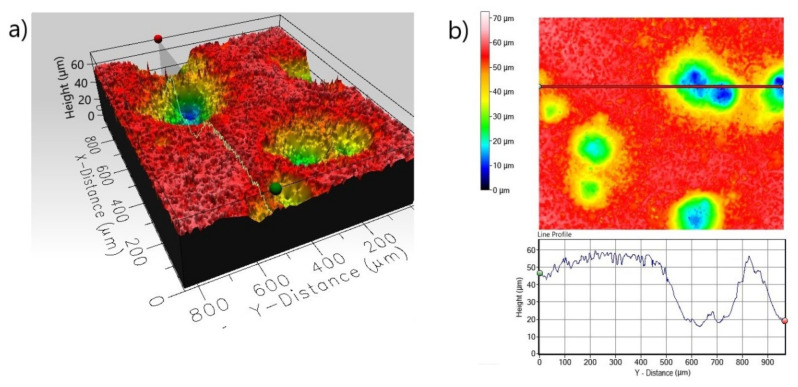
3D profile (**a**) and 2D profile (**b**) of the corroded surface of non-borided Nimonic^®^80A alloy after the corrosion resistance test in a 3.5% NaCl solution.

**Figure 15:**
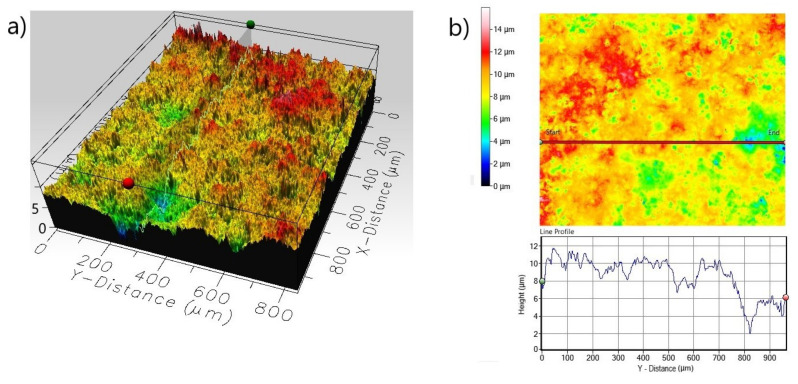
3D profile (**a**) and 2D profile (**b**) of the corroded surface of borided Nimonic^®^80A alloy after the corrosion resistance test in a 3.5% NaCl solution.

The 3D and 2D profiles of the corroded surface for the non-borided Nimonic^®^80A alloy are shown in Figure 14. The 3D profile (Figure 14a) reveals the presence of large diameter corrosion pits. It is clearly visible from the 2D and line profile (Figure 14b) that the diameter of a single corrosion pit exceeds 300 µm. Simultaneously, the maximum penetration depth of the single corrosion pit expressed as the maximum peak height *S_p_* was equal 72.591 µm. The 3D and 2D profiles of the corroded surface of borided Nimonic^®^80A alloy reveal the absence of deep corrosion pits (Figure 15). Moreover, the standard deviation *S_q_* of the distribution of surface heights in the surface area profile of the borided sample was equal 1.529 µm and was significantly lower than *S_q_* parameter measured for the non-borided sample (*S_q_* = 10.670 µm). However, on the basis of the 3D profile and line profile (Figure 15b), local lowering of the surface topography is visible in some areas. It could be a small corrosion pit with a depth not exceeding 8 µm.

The observation of surfaces after corrosion resistance tests can provide important information on the corrosion behavior of the non-borided and plasma paste borided nickel alloys. First, it should be noticed, that non-borided Nickel 201 was more resistant to a pitting corrosion attack than nickel-chromium alloys. In the case of nickel-chromium alloys (Inconel^®^600, Nimonic^®^80A), the cause of the corrosion pitting was the presence of differential corrosion micro-cells. It is generally known that a chromium content above 12% is sufficient to form a protective film consisting of chromium oxide (Cr_2_O_3_) on the surface of the nickel-based alloys. Simultaneously, this film is metastable (not fully stable), and may become active. The passive oxide film is not homogeneous because it includes some defects and impurities. Moreover, the passive oxide film is not homogeneous because it includes some defects and impurities. In addition, the microstructure of the substrate material (Inconel^®^600 or Nimonic^®^80A) consisted of different phases with different physical, chemical and electrochemical properties. Defects in the film (e.g., pores, cracks, imperfections, inclusions) are an easy way for penetration of chloride anions into substrate material. Chloride anions can destroy this film due to the more positive electrochemical potentials of some heterogeneous inclusions than pure nickel.

Pitting initiation occurs at localized locations on the defected metal surface, which may result from the oxide film failure, mechanical discontinuities, or microstructural phase heterogeneities, such as in the case of nickel-chromium alloys. In most cases, both the anodic and cathodic reactions continue inward in the gravity direction. For this reason, the bottom of the pits is rich in metal ions M^+^ due to the large number of anodic reactions. The reactions for nickel-chromium alloys are as follows:

Ni → Ni^2+^ + 2e^−^ (1)

Cr → Cr^2+^ + 3e^−^ (2)

In an aqueous electrolyte containing chlorine ions and oxygen molecules (e.g., 3.5% NaCl water solution) the Cl^−^ ions migrate towards the bottom of the pits and the O_2_ molecules react with water molecules on the metal surface. Therefore, metal chloride M^+^Cl^−^ and hydroxyl ions (OH)^−^ are produced [26,27]. This characteristic oxidation process is well known as a metal dissolution. Before forming the metal chloride, an aqueous compound is formed. In the case of nickel-chromium alloys the initial reactions are as follows:

O_2_ + 2H_2_O + e^−^ → 4(OH)^−^ (3)

Ni^2+^ + Cl^−^ → Ni^2+^Cl^−^ (4)

Then, metal chloride in a water-base electrolyte is hydrolyzed by water molecules, according to the reactions:

2Ni^2+^Cl^−^ + O_2_ + 2H_2_O → 2Ni(OH)_2_ + 2H^+^Cl^−^ (5)

2Cr^3+^Cl^−^ + O_2_ + 2H_2_O → 2Cr(OH)_3_ + 2H^+^Cl^−^ (6)

In the above reactions, H+Cl^−^ is the free hydrochloric acid that forms at the bottom of the pits, increasing the acidity at these locations. Therefore, at the bottom of the pits the hydrogen ion H^+^ concentration increases and the degree of acidity can be expressed as:

pH = −log[H^+^] (7)

Depending on the type of metal, different pH values can be encountered at the bottom of the pits. The values of pH calculated from the Nernst equation [28] was equal to 7.1 for Ni^2+^ reactions and 2.2 for Cr^3+^ reactions. Due to the lower pH of the chromium ions reactions, the susceptibility of nickel alloys with a high chromium concentration (Inconel^®^600, Nimonic^®^80A) to pitting corrosion was higher compared to pure nickel (Nickel 201).

The differences in corrosion resistance of layers produced after plasma paste boriding of nickel-based alloys were observed. The lowest *E_corr_* value was measured for the boride layer formed on Nickel 201, whereas, for borided layers formed on nickel-chromium alloys the corrosion potential was about 14% lower. The reason for this situation was the different phase composition of the produced borided layers. In the case of pure nickel, the borided layer contained only nickel borides (Ni_2_B, Ni_3_B, Ni_4_B_3_) [29,30]. Simultaneously, the formation of chromium borides (CrB, Cr_2_B) in the borided Inconel^®^600 and Nimonic^®^80A alloys resulted from high Cr content in those materials. The differences between the electrochemical properties of nickel borides and chromium borides caused the formation of micro-cells during the potentiodynamic polarization test in a 3.5% NaCl solution. The privileged areas of micro-cells formation were the areas in which the grains of nickel borides and grains of chromium borides bordered each other; consequently, the microstructure of the borided layer produced on nickel-chromium alloys was partialed to the anode region and cathode region. Therefore, during the potentiodynamic corrosion test micro-cells were formed between the different types of borides. The presence of such micro-cells was the reason for intensive corrosion of multicomponent borided layers, in which the anode was easy dissolved to the electrolyte.

## 4. Summary and Conclusions

The plasma paste boriding process was applied for the production of layers on nickel alloys with different chromium content. The nickel borides layer was formed on the Nickel 201 substrate. Whereas the high Cr content in Inconel^®^600 and Nimonic^®^80A alloys was the reason for obtaining a multiphase microstructure of the borided layers containing nickel and chromium borides. Based on the results obtained from the polarization curves, as well as based on the observations of the corroded surfaces, the following conclusions could be formulated:

1. Among all the non-borided samples, the highest corrosion resistance and the highest resistance to pitting corrosion were characteristic of a pure nickel sample.
2. The high chromium concentration in Inconel^®^600 and Nimonic^®^80A alloys resulted in high susceptibility of these alloys to pitting corrosion, as the presence of chromium ions caused a decrease in pH value at the bottom of the corrosion pits. Therefore, the intensive material dissolution was observed.
3. The electrochemical parameters *I_corr_* and *E_corr_* derived from the polarization curves indicated a higher corrosion resistance of all borided samples compared to the non-borided samples.
4. The highest corrosion resistance was obtained for plasma paste borided Nickel 201, due to the microstructure consisting only of nickel borides.
5. Differences between electrochemical properties of nickel borides and chromium borides caused the formation of micro-cells during the potentiodynamic polarization test of borided Inconel^®^600 and Nimonic^®^80A alloys; consequently, the intensive corrosion of the anodic regions occurred. Therefore, the corrosion resistance of borided layers formed on nickel-chromium alloys was lower when compared to the borided pure nickel.
6. Plasma paste boriding can be an effective barrier against corrosion in a 3.5% NaCl water solution.

## Figures and Tables

**Table 1:** Chemical composition of substrate materials (wt.%).

Elements and Material	Cr	Mn	Cu	Fe	Ti	Si	C	Al	Ni
Nickel 201	-	≤0.35	≤0.25	≤0.40	-	≤0.40	≤0.02	-	Balance
Inconel^®^600 Alloy	15.72	0.16	0.04	8.63	-	0.18	0.078	0.06	Balance
Nimonic^®^80A Alloy	19.52	≤0.01	0.01	0.25	2.55	0.09	0.085	1.44	Balance

**Table 2:** Electrochemical parameters *I_corr_* and *E_corr_*, determined based on the polarization curves.

Material	I_corr_[A/cm^2^]	E_corr_[V]
Non-Borided Nickel 201	9.1 × 10^−7^	−0.889
Borided Nickel 201	8.3 × 10^−7^	−0.853
Non-borided Inconel^®^600	9.8 × 10^−6^	−1.002
Borided Inconel^®^600	1.1 × 10^−6^	−0.953
Non-borided Nimonic^®^80A	9.7 × 10^−6^	−1.003
Borided Nimonic^®^80A	1.9 × 10^−6^	−0.902

**Table 3:** Roughness parameters of corroded surfaces after the corrosion resistance test.

Roughness Parameters Material	Line Roughness			Area Roughness			
	*R_a_* (µm)	*R_q_* (µm)	*R_p_* (µm)	*S_a_* (µm)	*S_q_* (µm)	*S_p_* (µm)	*S_z_* (µm)
Non-Borided Nickel 201	0.485	0.686	2.767	2.672	3.589	15.150	26.85
Borided Nickel 201	0.352	0.526	2.282	1.127	1.463	7.372	11.892
Non-Borided Inconel^®^600	1.121	1.484	5.147	7.779	10.812	20.441	58.144
Borided Inconel^®^600	0.253	0.336	2.074	1.191	1.534	8.209	16.880
Non-Borided Nimonic^®^80A	1.114	1.432	5.527	7.875	10.670	23.480	72.591
Borided Nimonic^®^80A	0.465	0.615	1.769	1.188	1.529	7.024	15.940
